# Structural and functional biomarkers of the insula subregions predict sex differences in aggression subscales

**DOI:** 10.1002/hbm.25826

**Published:** 2022-03-15

**Authors:** Haixia Long, Ming Fan, Qiaojun Li, Xuhua Yang, Yujiao Huang, Xinli Xu, Ji Ma, Jie Xiao, Tianzi Jiang

**Affiliations:** ^1^ College of Computer Science and Technology Zhejiang University of Technology Hangzhou China; ^2^ Institute of Biomedical Engineering and Instrumentation Hangzhou Dianzi University Hangzhou China; ^3^ School of Information Engineering Tianjin University of Commerce Tianjin China; ^4^ Zhijiang College Zhejiang University of Technology Hangzhou China; ^5^ Brainnetome Center and National Laboratory of Pattern Recognition Institute of Automation, Chinese Academy of Sciences Beijing China; ^6^ School of Artificial Intelligence University of Chinese Academy of Sciences Beijing China; ^7^ CAS Center for Excellence in Brain Science and Intelligence Technology Institute of Automation, Chinese Academy of Sciences Beijing China; ^8^ The Clinical Hospital of Chengdu Brain Science Institute, MOE Key Lab for Neuroinformation University of Electronic Science and Technology of China Chengdu China; ^9^ The Queensland Brain Institute University of Queensland Brisbane Queensland Australia

**Keywords:** aggression subscales, functional connectivity, gray matter volume, insula subregions, mediation analysis, sex

## Abstract

Aggression is a common and complex social behavior that is associated with violence and mental diseases. Although sex differences were observed in aggression, the neural mechanism for the effect of sex on aggression behaviors remains unclear, especially in specific subscales of aggression. In this study, we investigated the effects of sex on aggression subscales, gray matter volume (GMV), and functional connectivity (FC) of each insula subregion as well as the correlation of aggression subscales with GMV and FC. This study found that sex significantly influenced (a) physical aggression, anger, and hostility; (b) the GMV of all insula subregions; and (c) the FC of the dorsal agranular insula (dIa), dorsal dysgranular insula (dId), and ventral dysgranular and granular insula (vId_vIg). Additionally, mediation analysis revealed that the GMV of bilateral dIa mediates the association between sex and physical aggression, and left dId–left medial orbital superior frontal gyrus FC mediates the relationship between sex and anger. These findings revealed the neural mechanism underlying the sex differences in aggression subscales and the important role of the insula in aggression differences between males and females. This finding could potentially explain sexual dimorphism in neuropsychiatric disorders and improve dysregulated aggressive behavior.

## Graphical abstract

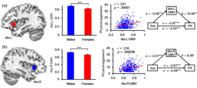

## 1 INTRODUCTION

Aggression is a general behavior in social interactions. Individuals with aggression may intend to cause physical and mental injury to others, especially when there is a conflict of interest between two or more individuals (Moyer, 1971; Nelson & Trainor, 2007). Aggression is a natural disposition of both humans and animals and is an adaptive trait during the early stages of human evolution (McCall & Shields, 2008). However, out‐of‐control aggression is pathological in modern society, while aggressive clinical symptoms are common in multiple neuropsychiatric disorders (Connor et al., 2019; Jensen et al., 2007). Therefore, aggression imposes a huge burden on individuals and society (Connor et al., 2019; Jensen et al., 2007; Nelson & Trainor, 2007).

Aggression is a complex social behavior and exhibits heterogeneity, such as physical violence or verbal arguments (Vitiello & Stoff, 1997). According to the Buss–Perry Aggression Questionnaire (one of the most widely used questionnaires of aggression), aggression consists of four subscales, including physical aggression, verbal aggression, anger and hostility, which represent motor, instrumental, emotional, and cognitive components, respectively (Buss & Perry, 1992). Previous reviews have shown that sex differences in aggression appear consistently (Archer, 2004; Hyde, 2014); however, the performances of males and females on different types of aggression were different. The sex differences between males and females are most prominent in physical aggression, followed by verbal aggression, hostility, and anger (Archer, 2004; Bacskai, Czobor, & Gerevich, 2011; Gerevich, Bacskai, & Czobor, 2007; Harris & Knight‐Bohnhoff, 1996; Kalmoe, 2015; Ramirez, Andreu, & Fujihara, 2001; Sadiq & Shafiq, 2020). The findings of studies about sex differences in hostility and anger were inconsistent; some studies found no sex differences in these subscales (Archer, 2004; Gerevich et al., 2007; Harris & Knight‐Bohnhoff, 1996; Sadiq & Shafiq, 2020), while other studies found significant differences (Bacskai et al., 2011; Menon, Sarkar, & Kattimani, 2015; Ramirez et al., 2001).

The insula is an important brain region involved in emotional processing and aggression (Blair, 2016; Nelson & Trainor, 2007; Phillips et al., 2004). Previous studies have demonstrated the effects of sex on the volume and functional connectivity (FC) of the insula, most of which showed that men have greater volume and FC of the insula than women (Dai et al., 2018; Hong et al., 2014; Jin et al., 2019; Klabunde, Weems, Raman, & Carrion, 2017; Lotze et al., 2019; Oz et al., 2021; Ruigrok et al., 2014; Sie, Chen, Shiau, & Chu, 2019; Wierenga, Langen, Oranje, & Durston, 2014). More interestingly, recent studies on sex classification have shown that the insula is one of the most discernable features to discriminate males from females (Brennan, Wu, & Fan, 2021; Weis et al., 2020). On the other hand, the insula was implicated in aggression. Reactive aggression provoked by other persons or the social environment was associated with insula‐related networks (Kramer, Riba, Richter, & Munte, 2011; Repple et al., 2017; White, Brislin, Sinclair, & Blair, 2014) and may cause a series of negative effects. A previous study investigated the neural mechanisms of reactive aggression and failed inhibition and found that the anterior insula played an important role in self‐control (Dambacher et al., 2015). Three related studies showed that cognitive regulation affected the activation and network of the insula in aggression (Abram et al., 2015; Achterberg, van Duijvenvoorde, Bakermans‐Kranenburg, & Crone, 2016; Jiang, Hou, Wang, & Li, 2018). Some studies investigated the influence of the social environment on aggression and found the activated anterior insula and ACC in response to social feedback and rejection (Achterberg et al., 2017; Achterberg, van Duijvenvoorde, van der Meulen, Bakermans‐Kranenburg, & Crone, 2018; Chester et al., 2014). Taken together, the insula plays an important role in aggression‐related networks. Based on histological or connectivity features, the insula can be divided into different subregions involved in various physiological functions (Cauda et al., 2012; Fan et al., 2016; Gordon et al., 2016; Kelly et al., 2012). Although previous studies have demonstrated that both the anterior insula and mid‐posterior insula are associated with aggression (Beames, Gilam, Schofield, Schira, & Denson, 2020; Cope et al., 2014; Cupaioli et al., 2021; Dambacher et al., 2015; Krauch et al., 2018), the effect of specific subregions within the insula on aggression subscales remains largely unclear. Therefore, it is of great interest to evaluate the neural mechanism for sex differences in aggression subscales by examining the association between the insula subregion and aggression subscales.

Since associations among sex, aggression subscales and insula were observed, the structure and function of the insula may underlie the neurobiological mechanism of the effect of sex on aggression subscales. However, the association among sex, insula, and aggression is still unclear, especially for different insular subregions. Therefore, this study aims to elucidate the role of insula subregions on the relationship between sex and aggression subscales in a large onefold Chinese young sample. We first subdivided insula into six subregions using the Brainnetome atlas (Fan et al., 2016) and further calculated each subregion's gray matter volume (GMV) and FC. The subtraits of aggression for each individual were assessed by the Buss–Warren Aggression Questionnaire. We further investigated the association between sex, aggression, GMV, and the FC map of each subregion in a large healthy Chinese sample.

## 2 MATERIALS AND METHODS

### 2.1 Subjects

This study included 360 right‐handed healthy Han Chinese young people. The board‐certified psychiatrists used the Structured Clinical Interview to screen all participants and ensured that they did not have Axis I disorders. All participants had no personal history or family history of psychiatric disorders or neurological diseases. Additionally, none of the subjects had any physical diseases, MRI contraindications, alcohol dependence, or drug addiction. The Ethics Committee of School of Life Science and Technology at the University of Electronic Science and Technology of China approved this study, and written informed consent was obtained from all participants.

Demographic information, including sex and age, was measured. The aggression of each participant was assessed by the Chinese version of the Buss‐Warren Aggression Questionnaire (AQ‐CV), including five subscales: physical aggression, verbal aggression, anger, hostility, and indirect aggression.

### 2.2 MRI data acquisition

All participants were scanned by a 3.0 Tesla GE MRI scanner (General Electric, Milwaukee, Wisconsin) and instructed to keep still and keep their eyes closed without falling asleep. After scanning, the subjects were asked to ensure that they were awake during the experiment. The structural MRI data were acquired by using the T1‐weighted brain volume (BRAVO) MRI sequence, and the parameters were as follows: repetition time (TR) = 8.16 ms, echo time (TE) = 3.18 ms, field of view (FOV) = 256 × 256 mm, flip angle = 7°, number of slices = 188, and voxel size = 1 × 1 × 1 mm. The gradient‐echo echo‐planar‐imaging (GRE‐EPI) sequence was used to obtain the resting‐state fMRI data with the following parameters: TR = 2,000 ms, TE = 30 ms, FOV = 240 × 240 mm, flip angle = 90°, matrix = 64 × 64, 255 volumes, 39 slices, and voxel size = 3.75 × 3.75 × 4 mm.

### 2.3 Resting‐state fMRI data preprocessing

The resting‐state fMRI data were preprocessed by using a pipeline BRAinNetome Toolkit (BRANT, https://github.com/YongLiuLab/brant-stable). The specific preprocessing steps included (a) discarding the first 10 volumes to maintain magnetization equilibrium; (b) slice timing; (c) head motion correction; (d) normalizing the EPI images to Montreal Neurological Institute (MNI) standard space with resampling to 3 × 3 × 3 mm; (e) regressing out head motion parameters, linear trends, white matter and cerebrospinal fluid signals; (f) temporal bandpass filtering between 0.01 and 0.08 Hz; and (g) smoothing with a Gaussian kernel of 6 mm full‐width at half maximum. The global brain signal variable is not included as a covariate in the regression model because this may exaggerate the negative correlation (Gotts et al., 2013; Saad et al., 2013). We excluded 28 participants since their maximum displacements were greater than 2 mm in any cardinal direction, such as *x*, *y*, *z*, or their maximum rotations were greater than 2° about any of the *x*–*z* axes.

### 2.4 GMV of insula subregions

The GMV map of each participant was produced using SPM12 (http://www.fil.ion.ucl.ac.uk/spm) which was executed in MATLAB R2016a (MathWorks, Natick, Massachusetts). First, the standard unified segmentation model was used to segment structural MRI images into gray matter and white matter. Furthermore, the gray matter template was constructed from the entire structural image dataset by the diffeomorphic nonlinear registration algorithm (DARTEL). Subsequently, the gray matter template was affinely registered to the tissue probability map in MNI standard space. Afterward, the gray matter image of each participant was nonlinearly normalized to the gray matter template in MNI space. Finally, modulation of the normalized gray matter images was carried out to obtain normalized and modulated gray matter map for each subject (termed GMV map).

**FIGURE 1:**
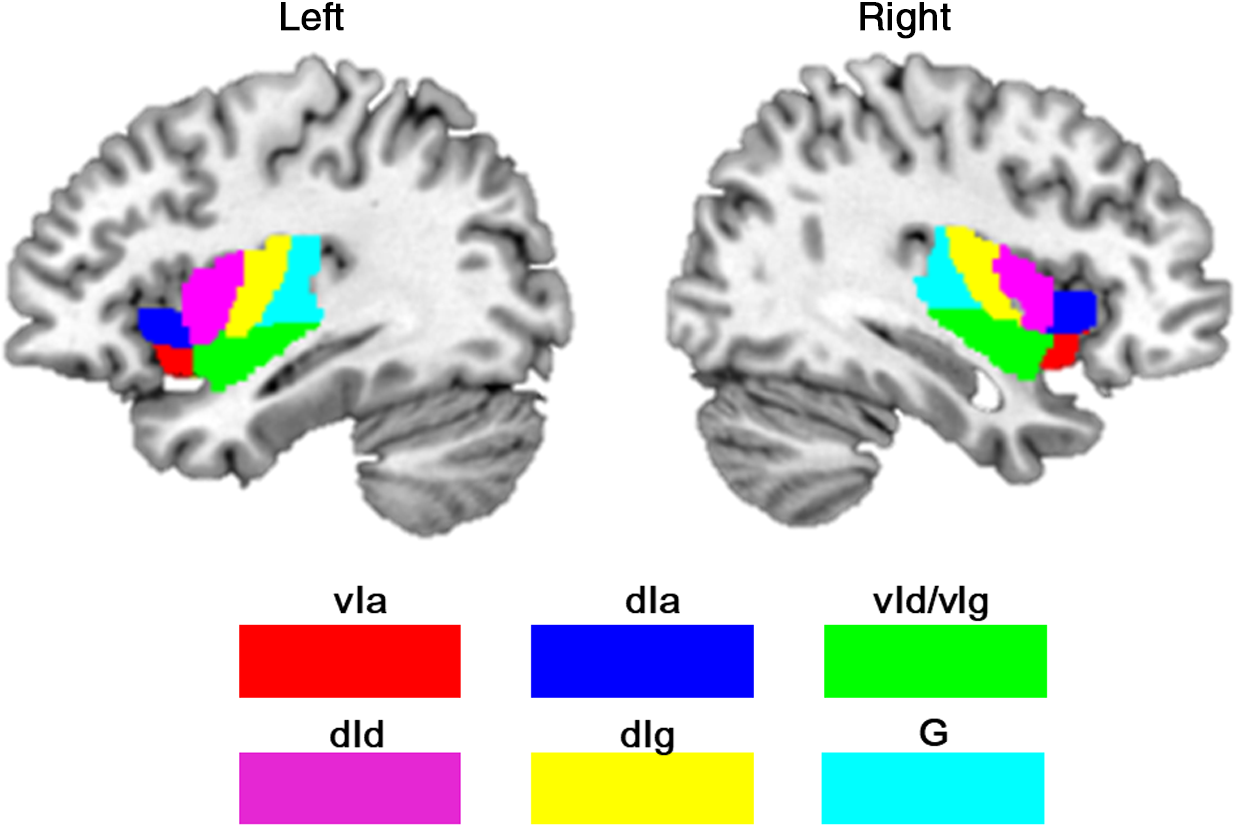
The anatomical location of 12 insula subregions. dIa, dorsal agranular insula; dId, dorsal dysgranular insula; dIg, dorsal granular insula; G, hypergranular insula; vIa, ventral agranular insula; vId_vIg, ventral dysgranular and granular insula

To divide the insula into subregions, the Brainnetome atlas (Fan et al., 2016) was implemented, which defines fine‐grained brain subregions using connection patterns. According to this atlas, the insula was segmented into six subregions in each hemisphere (Figure 1), including the hypergranular insula (G), ventral agranular insula (vIa), dorsal agranular insula (dIa), ventral dysgranular and granular insula (vId/vIg), dorsal granular insula (dIg), and dorsal dysgranular insula (dId). The above 12 subregions were extracted and resampled to 1.5 × 1.5 × 1.5 mm in MNI standard space. Afterward, the average GMV of each subregion was calculated for each participant.

### 2.5 Insula subregions FC analyses

The 12 subregions of the insula from the Brainnetome atlas were selected as the seed regions and resampled to 3 × 3 × 3 mm in MNI standard space. For each participant, Pearson correlation coefficients between the mean time series of each insula subregion and the time series of each voxel in the whole brain were calculated, resulting in 12 correlation maps. A Fisher *r*‐to‐*z* transformation was used to normalize the correlation coefficient (*r* value) to *z* scores. Subsequently, a one‐sample *t*‐test with a gray matter mask in SPM was carried out on whole *z* maps of all subjects for each insula subregion to test the significance of the connectivity between the seed region and the voxels of the whole brain. The clusters that survived at a threshold of *p* < .001 at a voxelwise false discovery rate (FDR) correction were determined to be significant and were used as binary masks for further analysis.

### 2.6 Statistical analysis

A two‐sample *t*‐test was performed to assess the effect of sex on age. This test was also used to evaluate sex differences in aggression subscales, such as physical aggression, verbal aggression, anger, hostility, and indirect aggression, with age as the confounding variable. The Bonferroni correction was used to control for the false discovery rate. All the above analyses were completed using SPSS24.

Sex differences in GMV of the 12 insula subregions were tested by two‐sample *t*‐tests with age as the confounding variable under Bonferroni correction. Then, correlation analysis was carried out to evaluate the association between GMV and aggression subscales while controlling for age. Third, the classic mediation model was used to determine whether the GMV of the insula subregions can serve as a potential mediator of the association between sex and aggression subscales, with sex as the independent variable, significantly correlated GMV as the mediator and significantly correlated aggression subscales as dependent variables, with age as the confounding variable.

The analysis process for the FC of insula subregions was similar to that for GMV. First, a two‐sample *t*‐test was used to assess the effect of sex on FC of each insula subregion in a voxel‐wise manner, while age was regarded as a nuisance covariate. The significance of sex differences was determined under a voxel‐level FDR corrected threshold of *p* < .001. The regions with significant sex differences (after FDR correction) were used for further analysis. The mean FC of each identified region was calculated. Then, the correlation between the mean FC of the identified insula subregions and aggression subscales was assessed with age as the nuisance covariate. Third, the mediation effect of FC of insula subregions on the association between sex and aggression subscales was estimated by the classic mediation model. In this model, sex was considered an independent variable, FC of insula subregions showing the significant correlation with aggression subscales was considered a mediator, and aggression subscales were considered dependent variables, while age was used as a confounding variable. The mediation analyses were conducted using the PROCESS v3.3 macro in SPSS24 with 5,000 bootstrap tests and 95% confidence interval (CI) (Hayes, 2012; Preacher & Hayes, 2008).

## 3 RESULTS

### 3.1 Demographics and behavioral characteristics

Statistical analysis showed no significant difference in age between males and females (Table 1). Significant effects of sex on physical aggression (*p* = 6.28 × 10^−7^), anger (*p* = .000001), and hostility (*p* = .009) were observed under Bonferroni correction. Specifically, males showed higher physical aggression and hostility scores but lower anger scores than females. Additionally, there was a trend of sex difference in verbal aggression (*p* = .02), in which a higher score was observed in the males than in the females (Table 1).

**TABLE 1:** Demographic characteristics of all participants

	Males	Females	Males vs. females *p* values
Sample size	186	174	–
Mean (SE)			
Age (years)^a^	19.01 (0.08)	18.92 (0.08)	.435
Physical aggression^b^	15.57 (0.37)	12.90 (0.38)	6.28 × 10^−7^
Verbal aggression^b^	11.66 (0.27)	10.77 (0.28)	.020
Anger^b^	14.54 (0.37)	17.21 (0.39)	.000001
Hostility^b^	17.80 (0.33)	16.56 (0.34)	.009
Indirect aggression^b^	13.28 (0.28)	13.74 (0.29)	.254

Abbreviation: SE, standard error.

^a^ Two‐sample *t*‐test was used to examine the sex difference in age.

^b^ Two‐sample *t*‐tests also were used to determine the effect of sex on the aggression subscales with age as the nuisance covariate.

### 3.2 Sex effect on GMVs of insula subregions

**FIGURE 2:**
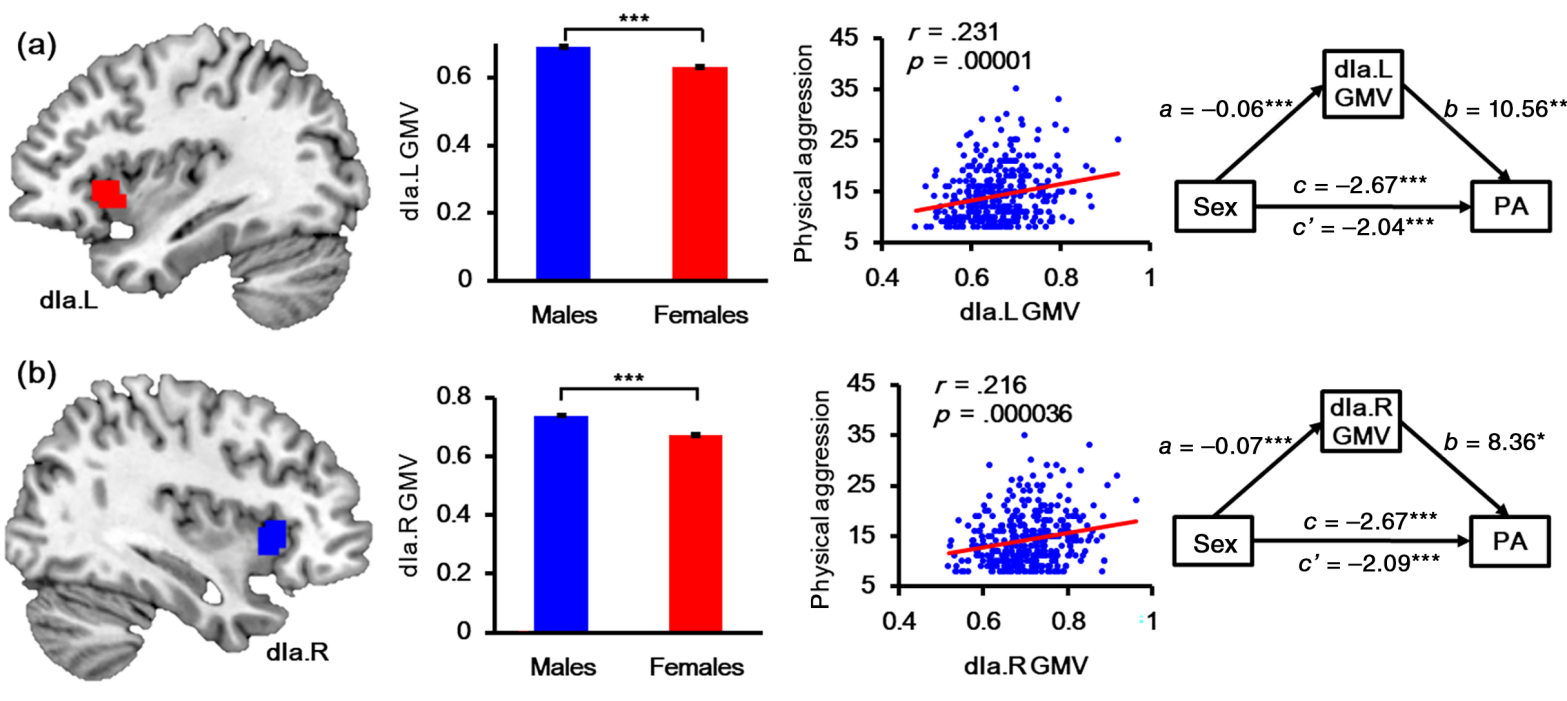
The results of gray matter volume (GMV) analyses of bilateral dIa. Part figure (a) shows the anatomical location of left dIa, the mean GMV of left dIa in males and females, the correlation between physical aggression and CMV of left dIa, and the mediation effect of GMV of left dIa on the association between sex and physical aggression from left to right. Part figure (b) shows the anatomical location of right dIa, the mean GMV of right dIa in males and females, the correlation between physical aggression and CMV of right dIa, and the mediation effect of GMV of right dIa on the association between sex and physical aggression from left to right. dIa, dorsal agranular insula; GMV, gray matter volume; L, left; R, right

This study demonstrates that sex significantly influenced the GMV of all insula subregions under the strict Bonferroni correction, as shown in Table 2. For all insula subregions, the males had higher GMV than the females. The physical aggression scores were significantly correlated with the GMV of left dIa (*r* = .231, *p* = .00001), right dIa (*r* = .216, *p* = .000036), and right dId (*r* = .185, *p* = .000417). The GMV of the left dIa and right dIa mediated the relationship between sex and physical aggression (Figure 2). The GMV of the left dIa significantly mediated the association between sex and physical aggression (Table 3), and the indirect effect was −0.6235. The bootstrapped 95% CI was [−1.1442 to 0.1606], accounting for 23.37% of the total effect. Additionally, GMVs of right dIa also significantly mediated the association between sex and physical aggression (Table 4), with an indirect effect of −0.5740, bootstrapped 95% CI [−1.1347 to 0.0729], accounting for 21.51% of the total effect.

**TABLE 2:** Sex difference in gray matter volume of insula subregions

	Males, mean (SE)	Females, mean (SE)	Males vs. females *p* values
vIa.L	0.633 (0.004)	0.594 (0.004)	3.56 × 10^−11^
vIa.R	0.706 (0.005)	0.650 (0.005)	8.52 × 10^−16^
dIa.L	0.689 (0.005)	0.630 (0.005)	2.35 × 10^−15^
dIa.R	0.739 (0.005)	0.671 (0.005)	1.39 × 10^−18^
vId/vIg.L	0.622 (0.004)	0.582 (0.004)	1.01 × 10^−10^
vId/vIg.R	0.654 (0.004)	0.601 (0.005)	2.62 × 10^−15^
dId.L	0.712 (0.005)	0.654 (0.005)	2.98 × 10^−14^
dId.R	0.695 (0.005)	0.637 (0.005)	2.54 × 10^−14^
dIg.L	0.659 (0.005)	0.617 (0.006)	9.99 × 10^−8^
dIg.R	0.669 (0.005)	0.609 (0.005)	8.40 × 10^−14^
G.L	0.627 (0.006)	0.572 (0.006)	2.29 × 10^−10^
G.R	0.591 (0.006)	0.537 (0.006)	4.35 × 10^−10^

*Note*: Two‐sample *t*‐tests were used to examine the sex differences in gray matter volume of insula subregions with age as a nuisance covariate.

Abbreviations: dIa, dorsal agranular insula; dId, dorsal dysgranular insula; dIg, dorsal granular insula; G, hypergranular insula; L, left; R, right; SE, standard error; vIa, ventral agranular insula; vId/vIg, ventral dysgranular and granular insula.

**TABLE 3:** Significant mediation effect of left dIa gray matter volume on the association between sex and physical aggression with age as a control variable

Step	Variables	Path	Coeff.	Boot SE	*t*	95% CI	
						LL	UL
1. (X → Y)	Sex, physical aggression	c	−2.67	0.52	−5.07***	−3.72	−1.64
2. (X → M)	Sex, dIa.L GMV	a	−0.06	0.01	−8.29***	−0.07	−0.05
3. (X + M → Y)	Sex, physical aggression	c′	−2.04	0.57	−3.59***	−3.19	−0.94
	dIa.L GMV, physical aggression	b	10.56	3.89	2.73**	2.92	18.52

*Note*: *p < .05, **p < .01, ***p < .001, two tailed.

Abbreviation: Boot SE, bootstrapping standard error; GMV, gray matter volume.

**TABLE 4:** Significant mediation effect of right dIa gray matter volume on the association between sex and physical aggression with age as a control variable

Step	Variables	Path	Coeff.	Boot SE	*t*	95% CI	
						LL	UL
1. (X → Y)	Sex, physical aggression	c	−2.67	0.52	−5.07***	−3.72	−1.64
2. (X → M)	Sex, dIa.R GMV	a	−0.07	0.01	−9.31***	−0.08	−0.05
3. (X + M → Y)	Sex, physical aggression	c′	−2.09	0.59	−3.59***	−3.26	−0.96
	dIa.R GMV, physical aggression	b	8.36	3.79	2.23*	1.08	15.90

*Note*: *p < .05, **p < .01, ***p < .001, two tailed.

Abbreviation: Boot SE, bootstrapping standard error; GMV, gray matter volume.

### 3.3 Sex effect on the FC of insula subregions

**FIGURE 3:**
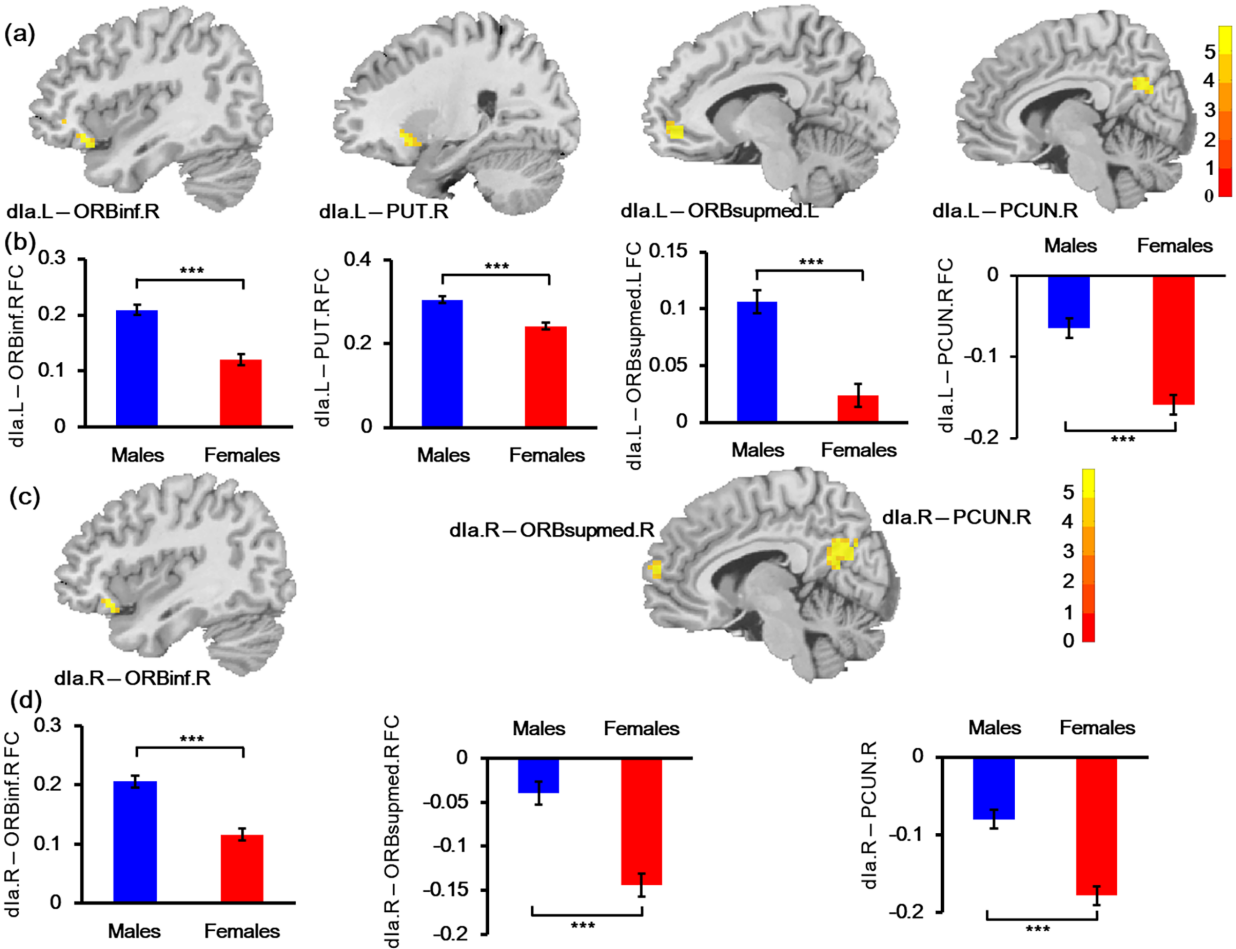
Sex effect on the functional connectivity (FC) of left dIa and right dIa. (a) The location of affected FC of the left dIa. (b) Mean FC strengths of each affected region with left dIa in males and females. (c) The location of affected FC of right dIa. (d) Mean FC strengths of each affected region with right dIa in males and females. dIa, dorsal agranular insula; FC, functional connectivity; L, left; ORBinf, orbital part inferior frontal gyrus; ORBsupmed, medial orbital superior frontal gyrus; PCUN, precuneus; PUT, putamen; R, right

**FIGURE 4:**
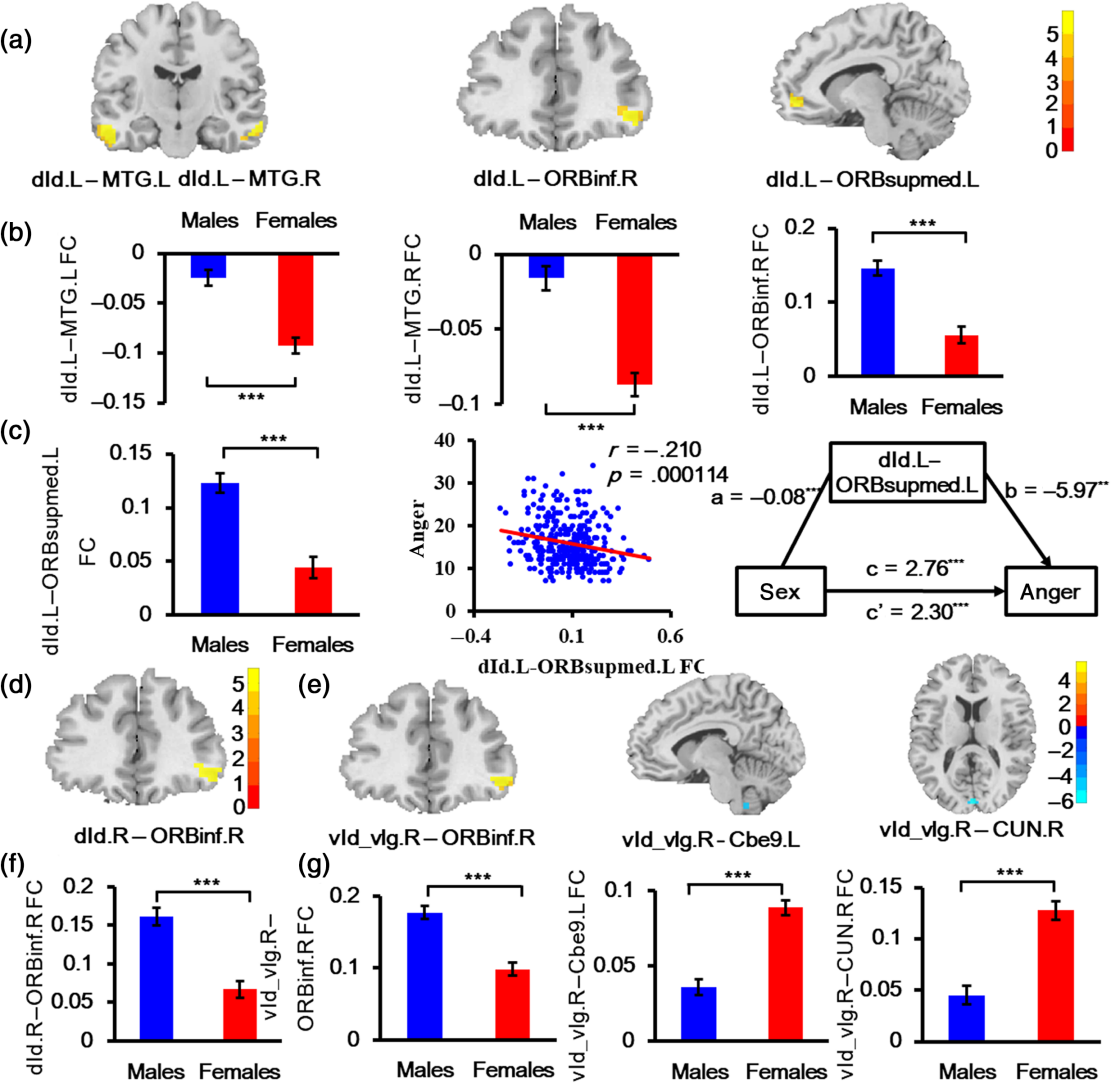
Sex effect on the functional connectivity (FC) of left dId, right dId, and right vId_vIg. (a) The location of affected FC of the left dId. (b) Mean FC strengths of dId.L–MTG.L, dId.L–MTG.R, and dId.L–ORBinf.R in males and females. Part figure (c) shows the mean FC strengths of dId.L–ORBsupmed.L in males and females, the correlation between left dId–left ORBsupmed FC and anger scores, the mediation effect of left dId–left ORBsupmed FC on the association between sex and anger from left to right. (d) The location of affected FC of the right dId. (e) The location of affected FC of right vId_vIg. (f) Mean FC strengths of dId.R–ORBinf.R in males and females. (g) Mean FC strengths of vId_vIg.R–ORBinf.R, vId_vIg.R–Cbe9.L, and vId_vIg.R–CUN.R in males and females. Cbe9, cerebellum 9; CUN, cuneus; dId, dorsal dysgranular insula; FC, functional connectivity; L, left; MTG, middle temporal gyrus; ORBinf, orbital part inferior frontal gyrus; ORBsupmed, medial orbital superior frontal gyrus; R, right; vId/vIg, ventral dysgranular and granular insula

Our study found the significant effect of sex on the FC of bilateral dIa, dId, and right vId_vIg (Table 5, Figures 3 and 4). Specifically, the FC between the left dIa and right orbital part inferior frontal gyrus (ORBinf), and right putamen (PUT), and left medial orbital superior frontal gyrus (ORBsupmmed), and right precuneus (PCUN) was higher in males than in females. While the results for right dIa FC were similar to those of left dIa FC, the FC of right dIa with three brain regions (right ORBinf, right ORBsupmmed, and right PCUN) was also higher in males. The connectivity of males between bilateral dId and right ORBinf increased compared with that of females. In addition, the left dId also showed other increased FC with the bilateral middle temporal gyrus (MTG) and left ORBsupmed. For vId_vIg, males also had increased FC between right vId_vIg and right ORBinf. On the other hand, right vId_vIg–left Cbe9 FC and right vId_vIg–right CUN FC decreased in males compared with females. Subsequent correlation analyses only found that the FC between the left dId and left ORBsupmed was negatively correlated with anger scores (*r* = −.210, *p* = .00014) under the Bonferroni corrected threshold of *p* < .05. Further mediation analysis confirmed that FC between the left dId and left ORBsupmed mediated the association between sex and anger. The indirect effect of sex on anger was 0.4668, making up 16.89% of the total effect, with a 95% CI [0.1092–0.8906] (Table 6 and Figure 4c).

**TABLE 5:** Sex affected functional connectivity of insula subregions

Seed regions	Abnormal regions	Peak MNI coordinate (*x y z*)	Peak intensity	Cluster size (voxels)
dIa.L	ORBinf.R	45 39 −9	5.2359	47
	PUT.R	27 18 −6	5.2555	32
	ORBsupmed.L	−6 48 −6	5.8535	43
	PCUN.R	6 −60 30	5.419	50
dIa.R	ORBinf.R	42 24 −15	5.4508	35
	ORBsupmed.R	3 63 15	5.6003	42
	PCUN.R	6 −69 27	5.7296	116
dId.L	MTG.L	−57 −15 −21	5.6649	57
	MTG.R	63 −12 −18	5.9344	35
	ORBinf.R	45 39 −9	5.5673	52
	ORBsupmed.L	−6 51 −6	5.7225	42
dId.R	ORBinf.R	57 33 −6	5.6034	49
vId_vIg.R	Cbe9.L	−6 −48 −51	−6.8828	67
	ORBinf.R	57 30 −6	5.9174	41
	CUN.R	3 −90 9	−7.023	48

*Note*: All the regions with cluster size ≥30 voxels survived at a threshold of corrected *p* < .001, FDR correction.

Abbreviations: Cbe9, cerebellum 9; CUN, cuneus; dIa, dorsal agranular insula; dId, dorsal dysgranular insula; L, left; MTG, middle temporal gyrus; ORBinf, orbital part inferior frontal gyrus; ORBsupmed, medial orbital superior frontal gyrus; PCUN, precuneus; PUT, putamen; R, right; vId_vIg, ventral dysgranular and granular insula.

**TABLE 6:** Significant mediation effect of FC between dId.L and ORBsupmed.L on the association between sex and anger with age as a control variable

Step	Variables	Path	Coeff.	Boot SE	*t*	95% CI	
						LL	UL
1. (X → Y)	Sex, anger	c	2.76	0.58	4.88***	1.62	3.90
2. (X → M)	Sex, dId.L–ORBsupmed.L FC	a	−0.08	0.01	−5.74***	−0.10	−0.05
3. (X + M → Y)	Sex, anger	c′	2.30	0.59	3.90***	1.14	3.45
	dId.L–ORBsupmed.L FC, anger	b	−5.97	2.20	−2.63**	−10.40	−1.69

*Note*: *p < .05, **p < .01, ***p < .001, two tailed.

Abbreviations: Boot SE, bootstrapping standard error; FC, functional connectivity.

## 4 DISCUSSION

This study mainly investigated the association between sex, structure, and function of the insula and aggression subscales. We identified significant effects of sex on physical aggression, anger and hostility. Sex also influenced the GMV and FC of insula subregions. Even more striking, the GMV of the left dIa and right dIa mediated the association between sex and physical aggression, and the left dId–left ORBsupmed FC mediated the association between sex and anger, which may reveal the underlying neural mechanism of sex differences in aggression subscales.

The observed significant effect of sex on the physical aggression is consistent with previous studies that showed males tended to take physical aggression action more than females (Archer, 2004; Gerevich et al., 2007; Harris & Knight‐Bohnhoff, 1996; Kalmoe, 2015; Sadiq & Shafiq, 2020). Additionally, we also found sex difference in hostility, that is, males having higher hostility than females, which is in line with a previous finding that males showed more hostility than females in Spanish and Japanese samples (Ramirez et al., 2001). These findings indicated that physical aggression may be associated with hostility. In addition, the higher anger scores in females than in males were also similar to the results in Isanzu and Buryats (Butovskaya et al., 2020). Additionally, an fMRI experiment of facial expressions also showed higher anger recognition levels in females than in males (Dores, Barbosa, Queiros, Carvalho, & Griffiths, 2020), which indicates their emotional dysregulation.

The insula is a heterogeneous brain region and is involved in various functions, such as emotion, cognitive control, attention, memory, perception, motor, and conscious awareness (Craig, 2009; Kurth, Zilles, Fox, Laird, & Eickhoff, 2010; Menon & Uddin, 2010; Uddin, Kinnison, Pessoa, & Anderson, 2014). This study used the Human Brainnetome Atlas to divide the insula into six subregions, including a dorsal anterior insula, a ventral anterior insula, a central region, a more ventral region, and two posterior subregions (Fan et al., 2016). In brief, the dorsal anterior insula is associated with cognitive function, the ventral anterior insula is associated with social–emotional tasks, and the mid‐posterior insula is related to interoception, perception, somatosensation, pain, and motor (Chang, Yarkoni, Khaw, & Sanfey, 2013; Kelly et al., 2012; Kurth, Eickhoff, et al., 2010; Kurth, Zilles, et al., 2010; Uddin, Nomi, Hebert‐Seropian, Ghaziri, & Boucher, 2017). Previous studies found that males exhibited significantly larger volumes across many cortex regions, including the insula, than females (Oz et al., 2021; Wierenga et al., 2014), which indicated sex differences in cortical development. Other studies have shown that sex affected the volumes of insula subregions. A related study demonstrated that males with posttraumatic stress symptoms had a larger volume of ventral anterior insula than females with posttraumatic stress symptoms, but this difference was not significant in control subjects (Klabunde et al., 2017). Another study found the larger GMV of the posterior insula in females than in males (Lotze et al., 2019). The inconsistent results of previous studies may be associated with different contexts of subjects and different locations of the insula. Therefore, our study investigated more fine‐grained insula subregions based on the Brainnetome atlas and found that males showed the larger GMV of each insula subregion than females, which was partly consistent with previous studies and revealed sex difference in brain maturation, with cortex volume decreasing more in females than males during puberty (Vijayakumar et al., 2016; Wierenga et al., 2014).

In addition, we also found the mediation of bilateral dIa GMV on the association between sex and physical aggression. The dIa belongs to the anterior insula and is related to cognitive tasks, decision making, and awareness (Craig, 2009; Deen, Pitskel, & Pelphrey, 2011). The anterior insula, which is involved in the salience network, is associated with social cognition and evaluation, and is sensitive to social saliency (Achterberg et al., 2016; Achterberg et al., 2018; Cacioppo et al., 2013). In addition, prior studies found that 19% of the variance in callous‐unemotional traits was explained by the GMV of the anterior insula in males, and callous‐unemotional traits were related to physical aggression (Raschle et al., 2018; Wright, Hill, Pickles, & Sharp, 2019). The fMRI studies also showed an association between anterior insula and reactive aggression and motor impulsivity (Chester et al., 2014; Dambacher et al., 2015; Werhahn et al., 2020). Therefore, compared with females, males received more social salience stimuli because of greater GMVs of bilateral dIa, which led to more physical aggression.

On the other hand, this study investigated the effect of sex on the FC of insula subregions. First, we found males showing greater FC between dIa and some prefrontal and parietal cortex, such as ORBinf, ORBsupmed, PCUN, and PUT, which was similar to previous studies (Hong et al., 2014; Sie et al., 2019). Additionally, there was significantly increased dId–MTG FC, dId–ORBinf FC and dId–ORBsupmed FC in males compared with females in our study, and the core affected regions are consistent with Dai et al.'s study (Dai et al., 2018). In addition, vId_vIg showed increased FC with ORBinf.R and decreased connectivity with Cbe9.L and CUN.R in males rather than in females, while a previous study found that women have greater FC between the posterior insula and cerebellum crus I (Sie et al., 2019), which was associated with autonomic regulation (Beissner, Meissner, Bar, & Napadow, 2013). Other studies considered the insula as a whole and found significant sex differences in FC between the insula and prefrontal cortex and sensorimotor cortex, where men showed increased FC in the insula than women (Jin et al., 2019). The effect of sex on FC mainly focuses on the relationship between the insula and brain regions in the default mode network (DMN) (Buckner, Andrews‐Hanna, & Schacter, 2008; Liu et al., 2010; Smith et al., 2009). Overall, compared with females, males demonstrated a stronger modulation of insula subregions in the DMN, while the modulation of vId_vIg on Cbe9 and CUN was weaker in males than in females for the compensation mechanism.

Moreover, correlation analysis and mediation analysis revealed the important role of left dId–left ORBsupmed FC in mediating the relationship between sex and anger. The dId belongs to the middle insula and is related to interoception, sensory perception, and somatosensation (Kelly et al., 2012; Kurth, Zilles, et al., 2010). The functional experiment showed that middle insula activity was associated with tolerance of anger expression (de Greck et al., 2012). Anger is a common experience during interpersonal communication, and some interpersonal conflict behaviors, such as unfair treatment and personal insults, may arouse anger (Averill, 1983; Baumeister, Stillwell, & Wotman, 1990; Gilam & Hendler, 2017). Moreover, anger is associated with emotion underregulation, and ORBsupmed is an important region of the emotion regulation network (Gilam & Hendler, 2017). Gilam et al.'s tDCS‐fMRI study validated the role of the ventromedial prefrontal cortex (vmPFC) in anger regulation (Gilam et al., 2018). Another study demonstrated that mPFC activity was positively correlated with self‐reported anger (Siep et al., 2019). Functional and effective connectivity analysis studies further illustrated that the FC of the mPFC and OFC was related to anger and proactive violence, and the effective connectivity among the insula, OFC and superior temporal gyrus was involved in anger processing (Eshel et al., 2021; Romero‐Martinez et al., 2019; Seok & Cheong, 2019). It is interesting to note the mediation effect of FC between left dId and left ORBsupmed on the relationship between sex and anger. The negative correlation between left dId–left ORBsupmed FC and anger indicated that the greater FC the subjects had, the stronger emotional regulation they showed, leading to less anger. Therefore, males showed stronger emotion regulation and less anger than females.

There are several limitations in the present study. First, our study only included Chinese samples to avoid stratification artifacts. Previous studies have shown that cultural background may influence aggression behavior (Butovskaya et al., 2020; Hyde, 2014; Ramirez et al., 2001). Therefore, further studies in different populations are needed to clarify the effect of sex on aggression in different populations. Second, our study only investigated the effect of sex on aggression and related neural mechanisms. In fact, aggression is a very complex social behavior that is influenced by multiple factors, such as genetic or environmental factors. Thus, further studies are needed to assess the effects of other factors on aggression. Advanced studies using machine learning models are also needed to predict aggression based on images and behavior measures. Third, although the Brainnetome atlas has been validated to effectively define more fine‐grained brain subregions and is consistent with other brain parcellation atlases (Fan et al., 2016), the impact of the potential interindividual variability of the insular subregions should also be investigated in further studies.

## 5 CONCLUSION

In conclusion, the current study assessed the neural mechanism of sex differences in aggression subscales based on the structure and function of insula subregions. We found that sex significantly influenced physical aggression, anger, and hostility. The GMV of all insula subregions and FC of dIa, dId, and vId_vIg were significantly different between males and females. More interestingly, this study found that the GMV of bilateral dIa mediated the association between sex and physical aggression, and the FC between the left dId and left ORBsupmed mediated the relationship between sex and anger. These findings reveal the neural mechanism underlying the sex differences in aggression subscales and could potentially be used to improve dysregulated aggressive behavior.

## CONFLICT OF INTEREST

The authors declare that they have no conflict of interest.

## Data Availability

The authors do not have permission to share raw data. The data that support the findings of this study are available from the corresponding author upon reasonable request.
